# Performance assessment of the 2 *γ*positronium imaging with the total-body PET scanners

**DOI:** 10.1186/s40658-020-00307-w

**Published:** 2020-06-30

**Authors:** P. Moskal, D. Kisielewska, R. Y. Shopa, Z. Bura, J. Chhokar, C. Curceanu, E. Czerwiński, M. Dadgar, K. Dulski, J. Gajewski, A. Gajos, M. Gorgol, R. Del Grande, B. C. Hiesmayr, B. Jasińska, K. Kacprzak, A. Kamińska, Ł Kapłon, H. Karimi, G. Korcyl, P. Kowalski, N. Krawczyk, W. Krzemień, T. Kozik, E. Kubicz, P. Małczak, M. Mohammed, Sz. Niedźwiecki, M. Pałka, M. Pawlik-Niedźwiecka, M. Pędziwiatr, L. Raczyński, J. Raj, A. Ruciński, S. Sharma, S. Shivani, M. Silarski, M. Skurzok, E. Ł. Stępień, S. Vandenberghe, W. Wiślicki, B. Zgardzińska

**Affiliations:** 1 Faculty of Physics, Astronomy and Applied Computer Science, Jagiellonian University, prof. Stanisława Łojasiewicza 11, Cracow, 30-348 Poland; 2 Department of Complex Systems, National Centre for Nuclear Research, Otwock-Świerk, 05-400 Poland; 3 INFN, Laboratori Nazionali di Frascati, Frascati, 00044 Italy; 4 Institute of Nuclear Physics PAN, Cracow, Poland; 5 Institute of Physics, Maria Curie-Skłodowska University, Lublin, 20-031 Poland; 6 Faculty of Physics, University of Vienna, Vienna, 1090 Austria; 7 High Energy Physics Division, National Centre for Nuclear Research, Otwock-Świerk, 05-400 Poland; 8 2nd Department of General Surgery, Jagiellonian University Medical College, Cracow, Poland; 9 Department of Physics, College of Education for Pure Sciences, University of Mosul, Mosul, Iraq; 10 Department of Electronics and Information Systems, MEDISIP, Ghent University-IBiTech, De Pintelaan 185 block B, Ghent, B-9000 Belgium

**Keywords:** PET, Positronium imaging, Total-body PET, Medical imaging

## Abstract

**Purpose:**

In living organisms, the positron-electron annihilation (occurring during the PET imaging) proceeds in about 30% via creation of a metastable ortho-positronium atom. In the tissue, due to the pick-off and conversion processes, over 98% of ortho-positronia annihilate into two 511 keV photons. In this article, we assess the feasibility for reconstruction of the mean ortho-positronium lifetime image based on annihilations into two photons. The main objectives of this work include the (i) estimation of the sensitivity of the total-body PET scanners for the ortho-positronium mean lifetime imaging using 2*γ* annihilations and (ii) estimation of the spatial and time resolution of the ortho-positronium image as a function of the coincidence resolving time (CRT) of the scanner.

**Methods:**

Simulations are conducted assuming that radiopharmaceutical is labeled with ^44^*Sc* isotope emitting one positron and one prompt gamma. The image is reconstructed on the basis of triple coincidence events. The ortho-positronium lifetime spectrum is determined for each voxel of the image. Calculations were performed for cases of total-body detectors build of (i) LYSO scintillators as used in the EXPLORER PET and (ii) plastic scintillators as anticipated for the cost-effective total-body J-PET scanner. To assess the spatial and time resolution, the four cases were considered assuming that CRT is equal to 500 ps, 140 ps, 50 ps, and 10 ps.

**Results:**

The estimated total-body PET sensitivity for the registration and selection of image forming triple coincidences (2*γ*+*γ*_prompt_) is larger by a factor of 13.5 (for LYSO PET) and by factor of 5.2 (for plastic PET) with respect to the sensitivity for the standard 2*γ* imaging by LYSO PET scanners with AFOV = 20 cm. The spatial resolution of the ortho-positronium image is comparable with the resolution achievable when using TOF-FBP algorithms already for CRT = 50 ps. For the 20-min scan, the resolution better than 20 ps is expected for the mean ortho-positronium lifetime image determination.

**Conclusions:**

Ortho-positronium mean lifetime imaging based on the annihilations into two photons and prompt gamma is shown to be feasible with the advent of the high sensitivity total-body PET systems and time resolution of the order of tens of picoseconds.

## Background

**Fig. 1:**
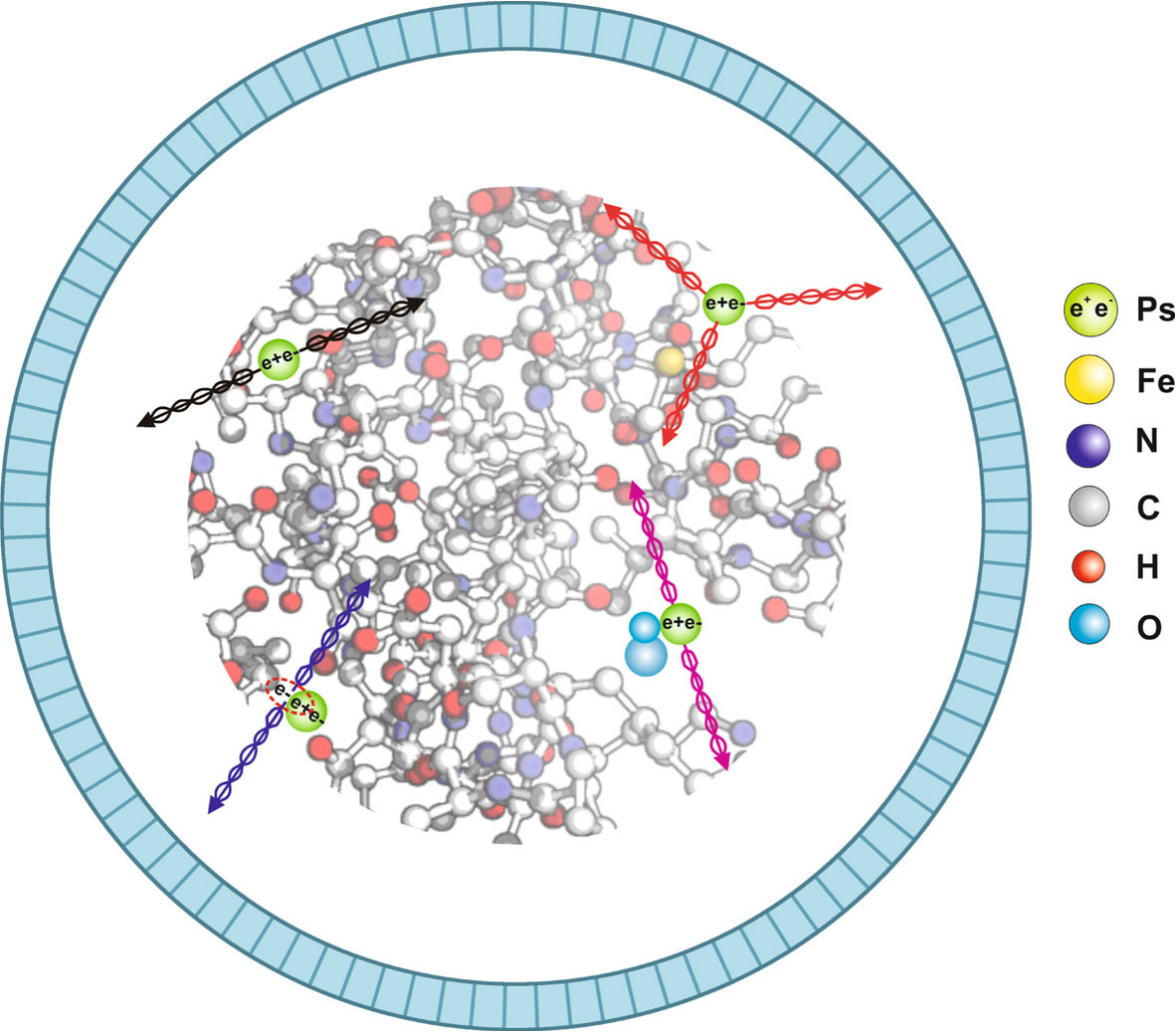
Pictorial representation of the single detection ring of the positron emission tomography scanner and (not to scale) magnified part of the hemoglobin molecule with pictorial representation of the possible ways of decays of positronium atoms (Ps) trapped in the intramolecular voids. Left-upper (black arrows) and right-upper (red arrows) indicate annihilations in the space free of electrons for para-positronium and ortho-positronium, respectively. Annihilation of positronium through the interaction with the electron from the surrounding molecule is shown in the left-lower corner (violet arrows) while in the right-lower part the conversion of ortho-positronium into para-positronium via interaction with the oxygen molecule and subsequent decay of para-positronium to two photons (magenta arrows) are presented [7]

In the positron emission tomography (PET), the density distribution of points of positron-electron annihilations, reflecting an image of metabolism rate of the radiopharmaceutical administered to the patient, is used for the diagnosis. The current PET technique does not take advantage of the fact that positron and electron may form a positronium atom. Yet, in up to about 40% cases [1, 2], positron-electron annihilations inside the human body proceed via the creation of the metastable positronium atom which in turn in quarter of cases appears as para-positronium (pPs) decaying to two photons and in three quarter of cases as ortho-positronium (oPs) decaying in vacuum into three photons. When trapped in the body, the ortho-positronium creation probability and mean lifetime strongly depend on the tissue’s nanostructure and the concentration of bio-active molecules (e.g., free radicals, reactive oxygen species, and antioxidants) which can interact with the emitted positrons as well as with the formed positronium [1]. The mean ortho-positronium lifetimes in the tissues varies from about 1.8 ns (as in pure water) to about 4 ns as measured for the human skin [3]. Whereas, the mean ortho-positronium lifetime differences for healthy and cancerous tissues are in the range of about 50 ps to about 200 ps [4–6]. In particular, ortho-positronium lifetime depends significantly on the size of free volume between atoms whereas its formation probability depends on their concentration. While both lifetime and formation probability depend on the concentration and type of biofluids and bio-active molecules [7]. Therefore, these ortho-positronium properties may be considered as diagnostic indicators complementary to the presently available SUV index [1, 8]. Recently, the in vitro studies indicated that indeed positronium mean lifetime and its production probability as well as the average time of direct annihilation differ for healthy and cancerous uterine tissues operated from the patients [4, 9]. Another in vitro measurements, performed with blood taken from patients before and after the chemotherapy or radiotherapy, demonstrated dependence of positronium properties in blood on the time after anti-neoplastic therapy [5]. In Fig. 1, an example of the hemoglobin molecule and the main mechanisms for the positronium annihilations inside the cells is presented.

The positron annihilation lifetime spectroscopy (PALS) is a well-established method in the material science [10–12]. However, in order to make use of the positronium properties in the in vivo medical diagnostics, development of the system combining PET and PALS is required [1, 7].

Recently, a method of positronium mean lifetime imaging, in which the lifetime, production probability, and position of positronium are determined on an event-by-event basis using *oPs*→3*γ* decay, was described [1, 8]. The place and time of ortho-positronium decay are reconstructed by the application of the trilateration technique [13, 14] which uses times and positions of registrations of three photons from *oPs*→3*γ* decay. The method is applicable for radiopharmaceuticals labeled with *β*^+^ decaying isotope emitting prompt gamma (e.g., ^44^Sc) [15, 16], where prompt gamma is used to determine the time of the ortho-positronium formation. The 3*γ* events were chosen because in case of the registration of the 3 *γ* in principle one can reconstruct the annihilation point with better spatial precision with respect to the case when only two photons are available. It was shown [1] that for the total-body J-PET scanner, when administering to the patient a typical activity of 370 MBq, one can expect about 700 registered events per cubic centimeter of the examined patient after 20 min of data collection. This is a promising result; however, the expected statistics is rather low. Low rate of *oPs*→3*γ* decays inside the tissue is due to the interaction of positronium with electrons from the surrounding atoms (pick-off process [17]) and due to the conversion [18–20] of ortho-positronium into para-positronium via interactions with the bio-active molecules (Fig. 1). Because of these processes, the ortho-positronium lifetime decreases in the tissue to the range of few nanoseconds [4, 9] and the fraction of its decay rate into three photons *f*_*oPs*→3*γ*_ decreases from *f*_*oPs*→3*γ*_=1 in vacuum to *f*_*oPs*→3*γ*_= *τ*_tissue_/*τ*_vacuum_ in the tissue [21], where *τ*_tissue_ and *τ*_vacuum_ denote the ortho-positronium mean lifetime in the tissue and in vacuum, respectively (*τ*_vacuum_=142 ns). For example, in case of *τ*_tissue_ = 2 *ns*, the ortho-positronium decays 70 times more frequent to 2 *γ* than to 3 *γ*.

Lifetime of the decaying object may be determined by the measurement of any of its decay channels, and hence, the ortho-positronium mean lifetime imaging can be performed based on the *oPs*→3*γ* decay [1], as well as based on the pick-off and conversion processes leading to the two back-to-back photons. In this article, we assess the feasibility of the 2*γ* ortho-positronium lifetime imaging for the total-body PET scanners assuming that the radiopharmaceutical is labeled with ^44^*Sc* isotope emitting positrons and prompt photon with energy of 1160 keV and using two back-to-back photons for the reconstruction of the ortho-positronium decay time and decay position. The prompt photon is used to determine the time of the creation of positronium. Reconstruction of the time difference between annihilation and emission of the positron enables to disentangle between processes when para-positronium decays to two photons (black arrows in Fig. 1) and ortho-positronium converts to two photons (magneta and violet arrows in Fig. 1). The lifetime of para-positronium (equal to 125 ps in vacuum) does not alter much as a function of properties of the tissues nanostructure (reaches about 230 ps) whereas the lifetime of ortho-positronium varies in the tissue in the range of few nanoseconds [4] and may occur to be useful as a diagnostic indicator [1, 7].

A statistical method of lifetime image reconstruction are yet to be conceived. Most recently, for the single detectors, 30 ps time resolution was achieved which is equivalent to position resolution of 4.5 mm along the line of response [22], and there is a continuous effort to improve it further even down to 10 ps [23, 24] which would enable to reconstruct the 2*γ* annihilation point along the LOR with precision of 1.5 mm. Such spatial precision of the reconstruction of annihilation point for each event would enable a direct reconstruction of the image as a density distribution of the reconstructed annihilation points. In such case, an iterative reconstruction procedures would not be needed and the spatial resolution of the image would be equivalent to the spatial resolution of the annihilation point reconstruction. Hence, the resolution of the ortho-positronium mean lifetime image will directly depend on the time resolution of the PET detector. At present, the newest TOF-PET scanners are characterized by the TOF resolution of about 210 ps [25] corresponding to the spatial resolution along the line of response (LOR) of about 3.8 cm. Recently, a detector design with SiPM has been reported, with CRT = 85 ps for 2×2×3 mm^3^ LSO:Ce doped with 0.4%Ca crystals and CRT of 140 ps for 2×2×20 mm^3^ crystals with the length as used in the current PET devices [26]. Thus, the TOF resolution, and consequently spatial resolution for a single event, is gradually improving by the development of new crystals, SiPMs [27], fast high frequency electronics [23], signal filtering [28] applications of the time ordered statistics [29, 30], signal waveform sampling [31, 32] including fast and cost-effective sampling in voltage domain [33], and advent of machine learning techniques [34].

In this context, it is worth mentioning that recently a new quenching circuit (QC) and single photon avalanche diode (SPAD) technology were introduced with 7.8 ps resolution [35] resulting in the resolution of 17.5 ps for the full chain of SiPM with QC and TDC [36].

In this article, based on the Monte Carlo simulations, we argue that with the time resolution in the order of tens of picoseconds and the advent of the high sensitivity total-body PET systems [37–39], the 2*γ* + *γ*_prompt_ mean lifetime positronium imaging based on time measurements may become possible in the future.

In the next section, the main assumptions applied in the simulations are presented. Further on, the sensitivity for the simultaneous registration of the back-to-back photons from positronium decay and prompt photon including selection of the image forming events is estimated for the total-body PET scanners built from LYSO crystal as well as for the cost-effective version of the total-body PET built from plastic scintillators. Next, the results of detailed Monte Carlo simulations of the response of the J-PET total-body scanner to the point-like sources arranged in the configuration as described in the NEMA norm are performed, and the regular PET 2 *γ* annihilation images as well as ortho-positronium mean lifetime images are reconstructed and compared for the four cases of assumed coincidence resolving time (CRT) of 500 ps, 140 ps, 50 ps, and 10 ps. Finally, it is shown that, owing to the large axial field-of-view of the total body scanners, the sensitivity for the positronium lifetime imaging is even larger than the present sensitivity for the 2*γ* metabolic imaging with the PET scanners having 20 cm axial length, even though the discussed positronium lifetime imaging requires registration of triple coincidence events including the prompt gamma and the two back-to-back photons.

## Materials and methods

**Fig. 2:**
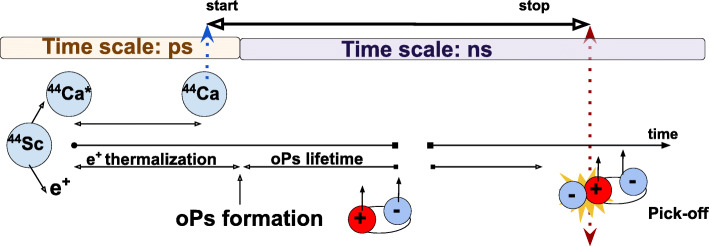
Scheme of the time sequence in the processes used for positronium imaging. A ^44^Sc nucleus undergoes *β*^+^ decay. Next, on the average after about 3 ps, excited ^44^Ca ^∗^ emits prompt gamma with energy of 1160 keV (dotted blue arrow). Parallelly, positron travels through matter, thermalizes, and forms an ortho-posironium bound state. Interaction with surrounding molecules or conversion process leads to emission of two photons. The mean ortho-positronium lifetime is in the order of nanoseconds, in contrast to the duration of thermalization [42] and deexcitation [43] processes which are in the order of 10 ps

Positronium mean lifetime imaging may be defined as spatially resolved determination of the ortho-positronium lifetime inside the patient’s body. For this purpose, it is required to determine the lifetime and position of ortho-positronium on the event-by-event basis, thus enabling calculation of the mean lifetime of ortho-positronium for each voxel of the image. The simulations presented in this article are conducted assuming that radiopharmaceutical is labeled with ^44^*Sc* isotope which emits positron and a remaining excited daughter nucleus ^44^*Ca*^∗^ emits prompt gamma with energy of 1160 keV via reaction chain: ^44^*Sc*→^44^*Ca*^∗^+*e*^+^+*ν*→^44^*Ca*+*γ*+*e*^+^+*ν* (see Fig. 2). The single (triple coincident) event used for imaging contains three signals that carry information about the position and time of the photons’ interaction in the detector: with one signal corresponding to prompt gamma and two signals corresponding to 511 keV photons originating from the annihilation of ortho-positronium while interacting with the molecular environment. The times and positions of interactions of 511 keV photons are used to determine the time and position of the annihilation point, whereas the time and position of the prompt gamma enable to determine the moment of the deexcitation of the ^44^Ca ^∗^ nucleus which with the precision of tens of picoseconds [40] can be associated with the moment of the creation of positronium. The lifetime spectrum determined for each voxel of the patient enables to extract information about the mean ortho-positronium lifetime and its production probability [8, 41].

### Analytic estimation of sensitivity

First, we performed simplified simulations in order to estimate a sensitivity for the registration and selection of triple coincidences useful for the positronium image reconstruction. In the simulation, it was assumed that the PET scanner is built from the cylindrical layer of scintillator detectors. Calculations were performed for the case of the LYSO scintillators with thickness of *d* = 1.81 cm as used in the EXPLORER [44] and plastic scintillators with thickness of *d* = 6 cm (two 3-cm-thick layers) as optimized for the J-PET total-body prototype [45]. The two back-to-back 511 keV annihilation photons and the 1160 keV prompt photon were emitted assuming that the activity is uniform along the 200-cm-long line source positioned in the central axis of the cylinder. For a single event, both annihilation photons and prompt gamma were emitted from the same point. The direction of back-to-back photons and the direction of the prompt gamma were generated independently, and in both cases, the isotropic distribution was assumed. The diameter of the cylinder was fixed to 80 cm whereas the length of the scanner was varied between 10 cm and 200 cm.

For a given axial field of view of the scanner, the sensitivity *S* was calculated as a function of the AFOV using a following formula:

1 $$\begin{array}{*{20}l} \int_{z=0}^{z=AFOV/2} dz \left[ \int_{\theta_{min}(z)}^{\theta^{a}_{max}(z)} (\epsilon_{det-a}(\theta) \cdot Att_{a}(\theta))^2 \cdot \sin\theta d\theta \right] \\ \left[ \int_{\theta_{min}(z)}^{\theta^{p}_{max}(z)} \epsilon_{det-p}(\theta) \cdot Att_{p}(\theta) \cdot \sin\theta d\theta \right] \end{array} $$

where *θ* denotes the polar angle between the photon’s direction of flight and the main axis of the tomograph. *L*_source_=200 cm denotes the length of the annihilation line source. The detection efficiency for annihilation photons *ε*_det−*a*_ and prompt gamma *ε*_det−*p*_ were calculated as (1−*e*^−*μ*·*d*/sin*θ*^), where *μ* denotes the attenuation coefficient which for 511 keV (1160 keV) photons was assumed to be *μ*=0.833 cm^−1^(0.413 cm^−1^) and *μ*=0.098 cm^−1^(0.068 cm^−1^) for LYSO and plastic scintillators, respectively. Att_*a*_(*θ*) and Att_*p*_(*θ*) indicate attenuation in the body of annihilation photons and prompt gamma, respectively. The influence of attenuation was estimated as $\phantom {\dot {i}\!}e^{-\mu _{\text {water}} \cdot R_{\text {phantom}}/\text {sin}(\theta)}$ approximating the body as a cylindrical phantom with radius of *R*_phantom_ = 10 cm filled with water with *μ*_water_ equal to 0.096 cm^−1^ and 0.066 cm^−1^ for 511 keV and 1160 keV photons, respectively. In the case of the LYSO scintillators, the selection efficiency *ε*_sel−*a*_ of image forming signals for 511 keV photons was estimated as a fraction of the photoelectric effect which is equal to 0.34, whereas for the plastic scintillators where 511 keV photons interact in practice only via Compton effect *ε*_sel−*a*_ was set to 0.44 [46] which results in a reduction of the scatter fraction to the level of ∼ 35*%* [47]. As regards the selection efficiency for the prompt photon with energy of 1160 keV which in both cases (LYSO and plastic) is registered predominantly via Compton effect (in ∼100% for plastics and 89% for LYSO), the selection efficiency *ε*_sel−*p*_ (both for LYSO and plastic scintillators) was equal to 0.66, just as a fraction of the energy deposition spectrum with deposited energy larger than the one from the 511 keV photons.

The above quoted values of attenuation coefficients and the fractions of photoelectric and Compton effects were extracted from the data base maintained by the National Institute of Standards and Technology [48].

### Detailed Monte Carlo simulations

The detailed studies of the spatial and time resolution of the mean lifetime positronium images as a function of the time resolution of the detector system were performed on the example of the cost-effective total-body PET built from plastic scintillators. Analogously as in reference [1], the ideal J-PET detector system consisting of four concentric cylindrical layers, filled with plastic scintillator strips, was simulated. The inner radius and axial field-of-view were chosen to be *R* = 43 cm and AFOV = 200 cm, respectively. For the single plastic strip, the cross section of 7×19 mm^2^ is chosen as it is used in the current version of the J-PET prototype [49].

In the plane comprising the central detector axis, the 1-mm radius cylindrical ^44^Sc sources surrounded with different materials were simulated. Their positions were chosen according to the NEMA NU 2-2012 norm [50], and for each position, a different mean lifetime of ortho-positronium was assumed, as it is listed in the Table 1. The values of the mean ortho-positronium lifetime were chosen in the range expected for ortho-positronium produced in the human body [1, 4].

**Table 1:** Coordinates of simulated point-like sources positioned according to the NEMA norm. Each source is characterized by a different mean lifetime of ortho-positronium

Position	Coordinates [cm]	Simulated oPs mean lifetime [ns]
1	(1, 0, 0)	2.0
2	(10, 0, 0)	2.4
3	(20, 0, 0)	2.8
4	(1, 0, 75)	2.2
5	(10, 0, 75)	2.6
6	(20, 0, 75)	3.0

For the studies presented in this article, 5×10^6^ events with the emission of prompt gamma from ^44^*Sc* decay followed by the creation of ortho-positronium and its subsequent decay into two photons were generated. Subsequently, for each generated event, the response of the idealized J-PET scanner was simulated and then the standard 2*γ* image as well as the mean lifetime positronium image were reconstructed. Standard PET image showing the density distribution of annihilation points was performed using TOF filtered back projection method (TOF-FBP) [51], and the mean lifetime positronium image is reconstructed by the determination of the annihilation point and ortho-positronium lifetime on the event by event basis. Positronium lifetime image is constituted from mean ortho-positronium lifetimes determined for each image voxel.

The simulations were conducted in the following steps: For each event (i) the position of the annihilation was generated, in the arrangement required by the NEMA [50] norm (Table 1); (ii) the prompt photon with energy of 1160 keV was emitted isotropically; (iii) in each event, a creation of ortho-positronium atom was assumed and its decay time was generated with the exponential probability density distribution assuming mean lifetime depending on the position (see Table 1); (iv) for each event, an ortho-positronium annihilation (pick-off or conversion processes) into two 511 keV back-to-back photons was generated assuming the isotropic emission independent of the emission of the prompt gamma; (v) hit-positions and energy depositions of annihilation photons and prompt gamma in the J-PET scanner were simulated taking into account cross sections for Compton interactions of gamma photons in plastic scintillators; (vi) the experimental hit-position resolutions (axial and radial) was accounted for by smearing the generated positions with Gaussian functions having 5 mm (FWHM) as expected for J-PET scanner with the assumed SiPM and WLS readout [1, 47]; (vii) the time resolution was included by smearing the generated interaction times with Gaussian distributions corresponding to CRT values of {500,140,50,10} ps (the values after smearing will be referred to as *registered*); (viii) for each *registered* event, the time and position of annihilation point as well as the time of the emission of prompt gamma was reconstructed; and finally (ix) for each voxel of the image, a mean ortho-positronium lifetime was reconstructed.

The simulation methods for the above listed steps were described in details in the previous publications [1, 30, 46, 52, 53].

## Results

**Fig. 3:**
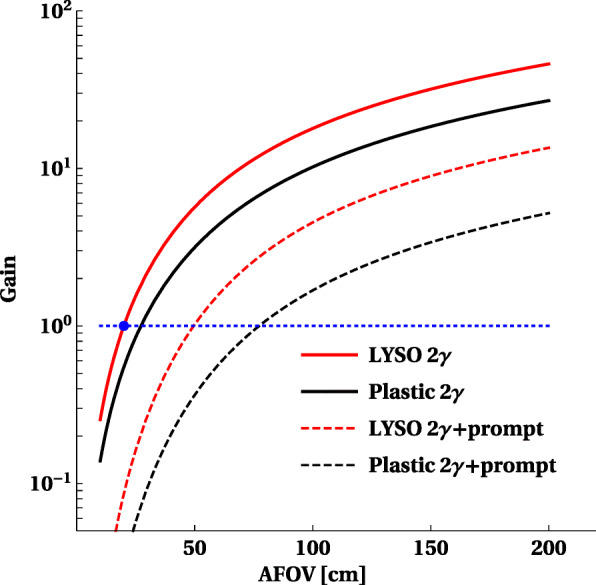
Relative gain in sensitivity *S* as a function of the AFOV of the scanner. The gain is calculated relative to sensitivity for the standard 2*γ* imaging using LYSO PET with AFOV = 20 cm (blue dot). The gains for 2*γ* (solid lines) as well as for 2*γ*+*γ*_prompt_ (dashed lines) are shown for LYSO and plastic scintillators as indicated in the legend. The red and black color indicate result for the LYSO and plastic scintillators, respectively

Figure 3 presents the gain of the sensitivity calculated as defined by (Eq. 1) in the “Materials and methods” section. The gain is calculated with respect to the sensitivity of the PET from LYSO crystals with AFOV = 20 cm for the standard 2*γ* imaging (indicated as blue dot in the figure). The relative gain is presented as a function of AFOV of the scanner. Dashed lines indicate that for the (2*γ*+*γ*_prompt_) triple coincidences with AFOV = 200 cm, the achievable gain is equal to factor of about 13.5 and 5.2 for LYSO and plastic-based PET, respectively. It indicates that the 2*γ* +*γ*_prompt_ positronium imaging is feasible with the total-body PET scanners with sensitivities even higher than the one of the current PET with AFOV ∼20 cm. For the comparison, in order to cross-check the estimations, the gain for the standard 2*γ* imaging is shown. The red and black solid lines indicate sensitivity gain expected for the registration of the image forming events with two back-to-back annihilation photons (2*γ*) calculated for the LYSO and plastic-based scanners, respectively. As expected, [54, 55] for the whole body scan with total-body LYSO PET the sensitivity is increased by more than factor of forty. The black solid line indicates that for the whole-body scan, the total-body PET from plastic scintillators also provides a large, about twentyfold, gain in sensitivity.

**Fig. 4:**
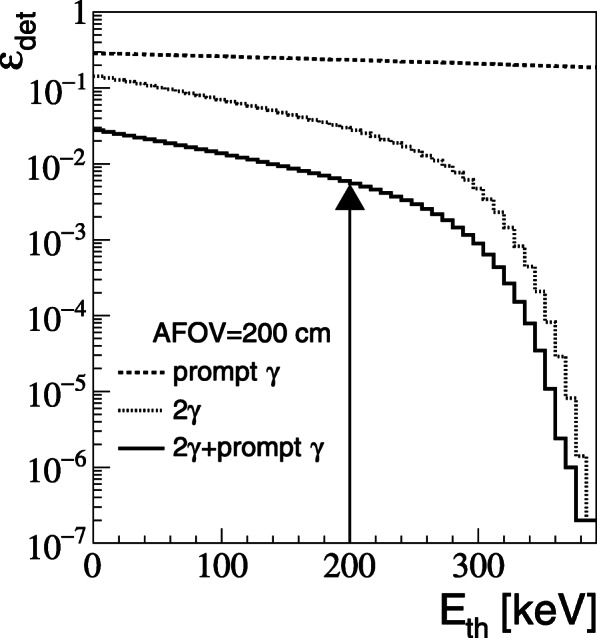
Registration efficiency (taking into account the geometrical acceptance, probability of gamma quanta registration in the plastic scintillator and J-PET resolution) as a function of applied threshold for the cases of prompt gamma (dashed line), two back-to-back 511 keV photons (dotted line), and two photons simultaneously with the prompt gamma with energy loss higher than 400 keV (solid line)

Figure 4 presents sensitivities (detection efficiency) calculated using detailed Monte- Carlo method (the “Materials and methods” section) for the case of the total-body plastic PET with AFOV = 200 cm and total plastic thickness of 6 cm. The dashed line indicates overall geometrical and detection efficiency for the registration of the prompt gamma (1160 keV), and the dotted histogram shows detection efficiency for the registration of the back-to-back 511 keV photons. The results are shown as a function of the energy deposition threshold. Solid line indicates overall efficiency for the registration of back-to-back photons with coincident detection of prompt photon with energy deposition larger than 400 keV. Such condition enables to disentangle prompt gamma from 511 keV photons. In order to decrease the scatter fraction down to 35%, a minimum energy deposition of 200 keV is required for the 511 keV photons [47, 53, 56]. Therefore, for the 2*γ* positronium mean lifetime imaging of the 200-cm long objects, with the plastic total-body PET, a total detection and selection efficiency of about 0.5% are expected (as indicated in Fig. 4 by an arrow). Thus, with the plastic total-body PET about 6.6·10^8^ image forming triple coincidence events (2*γ*+*γ*_prompt_) may be collected assuming (i) a 20-min scan according to the standard whole-body protocol, (ii) activity of 370 MBq (10 mCi) administered to the patient [55], (iii) detection sensitivity of 0.5*%*, and (iv) 30% of fraction of positron annihilations through the formation of ortho-positronium atoms in the body. This corresponds to about 10^4^ event forming events per cubic centimeter for the plastic-based total-body PET. While, when taking into account the ratio between dashed lines in Fig. 3, 2.6·10^4^ events per cubic centimeter are expected for LYSO-based total-body PET. These numbers will be used later on to estimate mean lifetime resolution for positronium imaging.

**Fig. 5:**
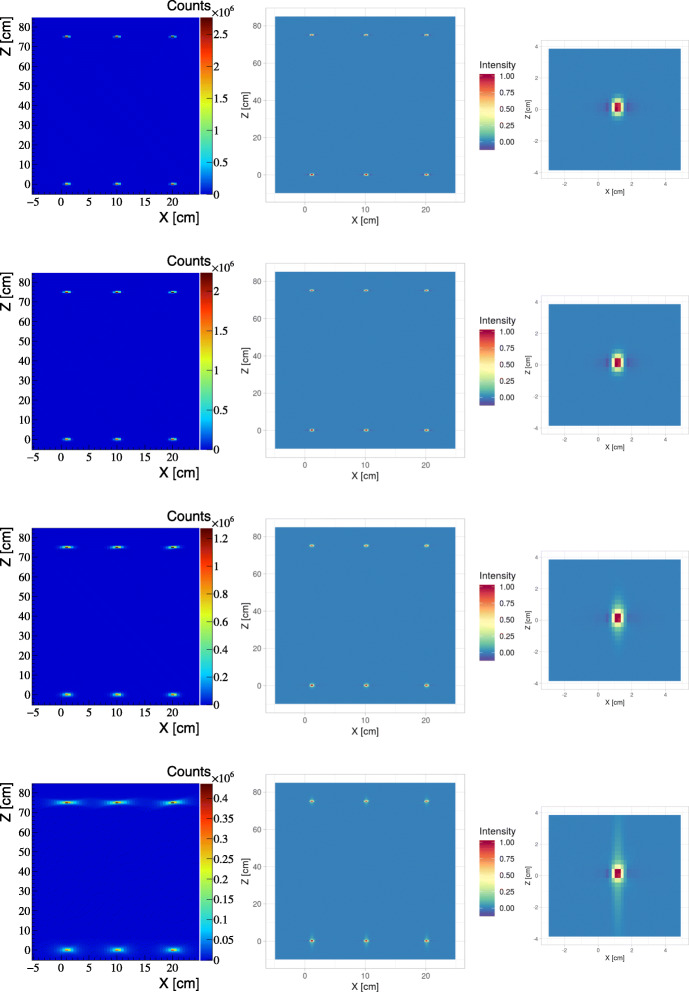
**Left:** Reconstructed distribution of annihilation point spatial coordinates. The voxel size is equal to 5×5×5 mm^3^. **Middle:** Reconstructed image of six sources obtained while applying the TOF-FBP algorithm. The voxels: 1.8×1.8×2.9 mm^3^. **Right:** Reconstructed source placed at (*x*,*y*,*z*)=(1,0,0) cm. Each row show results with different resolution: CRT =10 ps (top row), CRT =50 ps (second row), CRT =140 ps (third row), and CRT =500 ps (bottom row)

Figure 5 compares standard PET images determined for the point-like sources arranged according to the NEMA norm recommendations (Table 1). The coronal *XZ* cross-section along *y*=0 cm are shown. Left column shows images obtained directly as a density distribution of annihilation points assuming TOF resolution of CRT = 10 ps (top row), CRT = 50 ps (second row), CRT = 140 ps (third row), and CRT = 500 ps (bottom row). Middle column indicates images determined using the TOF-filtered back projection (TOF-FBP) algorithm, and the right column presents enhanced view of images of the source close to the center of the scanner. The obtained point spread functions (PSF) are listed in Table 2. The result indicates that already for CRT = 50 ps, the PSF of the direct image is comparable with the PSF achievable when applying reconstruction with TOF-FBP algorithm.

**Table 2:** Point Spread Function (PSF) obtained for the direct and TOF-FBP images as a function of CRT. Values presented are for position (*x*=20,*y*=0,*z*=75) cm

	PSF direct image [mm]		PSF TOF-FBP image [mm]	
CRT [ps]	Radial	Axial	Radial	Axial
10	4.2	4.8	4.5	5.5
50	5.4	5.2	4.5	5.5
140	10.2	6.8	5.0	6.0
500	29.9	6.8	5.0	6.0

**Fig. 6:**
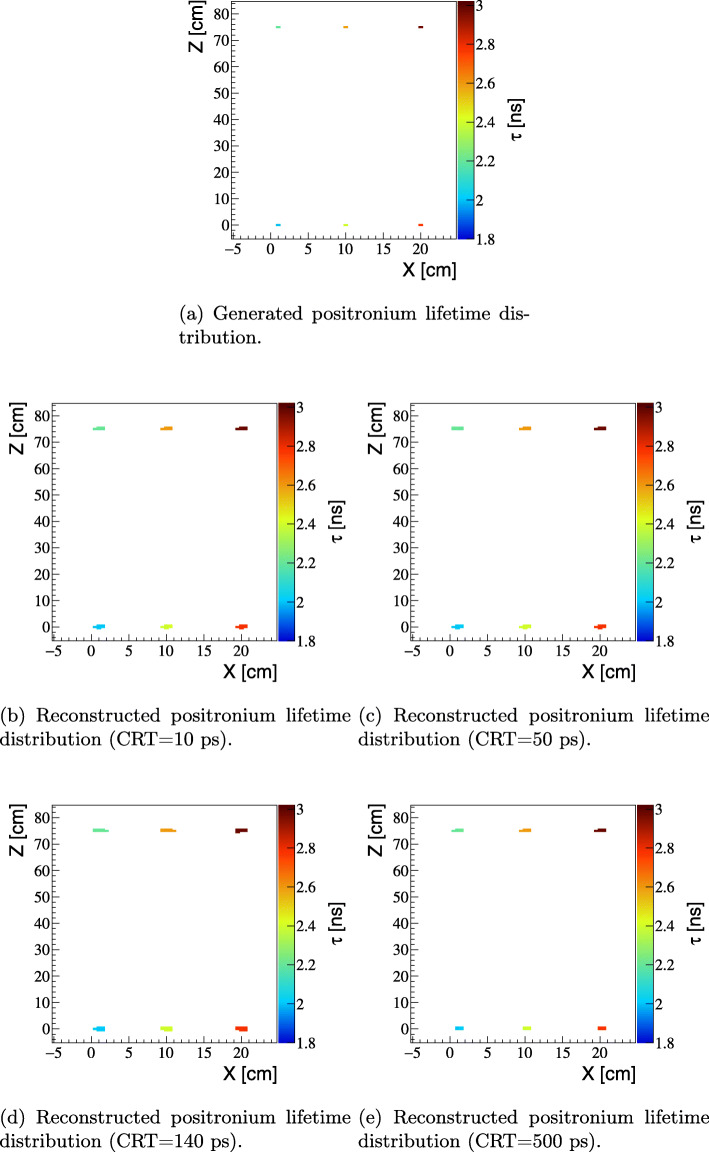
Distributions of generated positronium lifetimes „ and reconstructed ones, assuming the CRT value of 10 ps (**b**), 50 ps (**c**), 140 ps (**d**) and 500 ps (**e**). The voxel size is equal to 5×5×5 mm^3^

**Fig. 7:**
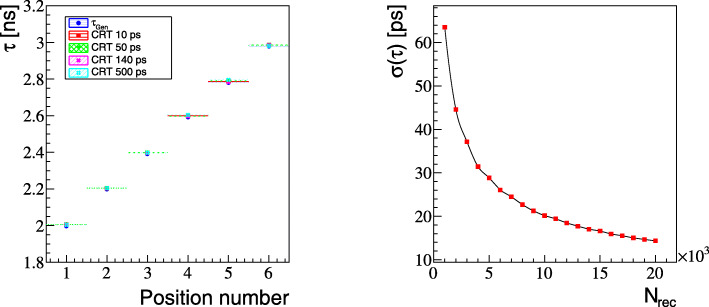
**Left:** Comparison between generated positronium mean lifetime and reconstructed one assuming different detector CRT resolutions as a function of NEMA position number (see Table 1). Differences between obtained results are in the order of $\mathcal {O}$(10 ps). **Right:** Resolution of the mean lifetime determination as a function of detected entries in a single voxel

Finally, the results for the mean lifetime positronium image are presented. The triple coincidence events (2*γ*+*γ*_prompt_) were simulated and analyzed as it is described in the “Materials and methods” section. When using events with triple coincidences 2*γ*+*γ*_prompt_, it is possible to reconstruct position and time of the creation of two back-to-back photons and the time of the creation of the positron. The latter can be deduced based on the time and position of interaction of the prompt gamma and the position of the annihilation point. Thus, for each voxel of the image, a spectrum of differences between the time of annihilation and the time of positron emission can be created enabling mean lifetime ortho-positronium reconstruction. Figure 6 compares ortho-positronium mean lifetimes images reconstructed based on simulated signals (described in the “Materials and methods” section) to the generated image. The simulation and reconstruction was performed for four cases assuming time resolution of the scanner to be CRT = 10 ps, 50 ps, 140 ps, and 500 ps. All reconstructed images reflect the generated image very well. The quantitative comparison is shown in the left panel of Fig. 7. Figure shows that for each simulated source position, the reconstructed mean lifetimes agree with the generated values within about 10 ps. The resolution of the reconstructed mean lifetime depends on (i) the detector’s time and spatial resolution, (ii) on the mean ortho-positronium lifetime (*τ*_tissue_), as well as (iii) on the number of events assigned to a given voxel. The right panel of Fig. 7 presents the resolution as a function of the number of events. The result indicates that a resolution of better than 20 ps (RMS) is achievable for the number of image forming events (10^4^) expected per cubic centimeter in a 20-min total-body scan. The result is valid for all tested CRT values since they are much smaller than the mean lifetime of ortho-positronium atoms in the body (few nanoseconds), and therefore, the reconstruction resolution of the mean lifetime depends predominantly on *τ*_tissue_ and may be approximately estimated as $\tau _{\text {tissue}}/\sqrt {N}$, where *N* denotes the number of events in a given voxel. It is important to stress that in Fig. 4, the values of sensitivities do not include losses in the number of events due to attenuation and scattering of photons in the patient. Inclusion of attenuation would decrease the number of positronium-image forming events by about an order of magnitude. Therefore, at the present early stage of the development of this method, it would be appropriate to strive to achieve first experimental images with larger voxel’s size (e.g., 2 × 2 × 2 cm^3^) ensuring the statistics of (10^4^) events per voxel.

## Discussion

Recently, it was reported that the mean lifetime of ortho-positronium, which is copiously produced in the body during the PET investigation, may be used as the in vivo diagnostic indicator complementary to the currently available SUV index [1, 7]. In this article, we assess the feasibility of ortho-positronium mean lifetime imaging using 2*γ* decays which occurs in the tissue due to the pick-off and ortho- to para-positronium conversion processes. We have shown that with the advent of the total-body PET and the improvement of time resolution to tens of picoseconds, the 2*γ* ortho-positronium mean lifetime imaging may be performed with the sensitivity and spatial resolution comparable with the current standard 2*γ* metabolic imaging obtainable by PET scanners with AFOV of about 20 cm. We also have shown that having the time resolution of tens of picoseconds, the 2*γ* mean lifetime positronium imaging becomes feasible for the cost-effective total-body J-PET scanner built from plastic scintillators. Calculations were preformed taking into account ^44^*Sc*-labeled radiopharmaceutical emitting prompt gamma with the energy of 1160 keV. In the calculations of the sensitivity gain, the attenuation of both two 511 keV annihilation and prompt photons in the 20-cm-diameter water phantom was taken into account. Quantitative estimations show that for the whole-body scan, the overall sensitivity for registration and image-forming selection of 2*γ*+*γ*_prompt_ events exceeds the sensitivity of current PET 2*γ* metabolic imaging twelvefold in case of total-body PET based on LYSO scintillators and fourfold in case of the total-body PET from plastic scintillators. Moreover, the obtained result (Fig. 3) indicates that the sensitivity for ortho-positronium mean lifetime image is becoming comparable with current PET sensitivities for AFOV of about 56 cm (LYSO PET) and 87 cm (plastic PET). Finally, the mean lifetime resolution achievable with the presented method for total-body PET is ranging between 10 ps and 20 ps for LYSO and plastic PET systems and it is predominantly due to the large (few ns) ortho-positronium mean lifetime while it is fairly independent of about order of magnitude smaller CRT values.

Positronium mean lifetime imaging with two photons, discussed in this article, is beneficial in relation to imaging using three photons due to the smaller attenuation of photons in the body and higher detection sensitivity. In case of the ortho-positronium 2*γ* imaging, the attenuation of photons in the body will be much smaller than for the *oPs*→3*γ* imaging. This is because in the latter case, there is one more photon which needs to escape from the body and the energy of photons in case of *oPs*→3*γ* ranges from 0 to 511 keV, and thus, these photons having energy lower than 511 keV are on the average more strongly absorbed in the body with respect to 511 keV photons from the 2*γ* annihilations. Moreover, the ortho-positronium mean lifetime imaging based on the *oPs*→2*γ* events may directly be applied in the present TOF-PET systems. Making the application of the proposed method possible in the future.

## Conclusions

The method discussed in this article, in general, enables to determine a spectrum of positron lifetime on a voxel by voxel basis. Allowing to determine not only an image of the mean lifetime of ortho-positronium (or in general a distribution of mean ortho-positronium lifetimes [41]), but it also gives access to the images of mean lifetime of the direct electron-positron annihilations and the probability of the positronium formation [8]. Establishing correlations of these parameters with the cancer grade requires systematic long-term study. There are first in vitro measurements indicating differences in positronium properties in healthy and cancerous tissues [4]. The application of the method advocated in this article makes an in vivo investigations possible which, due to the fact that positronium interacts in the living organisms with bio-active molecules, may reveal yet unknown features useful for diagnosis.

Finally, it is worth noting that one of the important features of the mean ortho-positronium lifetime image is that it does not require attenuation corrections [8]. This is because of the lifetime of ortho-positronium is independent of photons attenuation, and though suppression of photons in the body affects the statistics in a given voxel, it does not affect the shape of the lifetime spectrum, leaving the mean lifetime unaltered.

## Data Availability

The data that support the findings of this study are available from the corresponding author upon request.
